# Acute pathophysiological myocardial changes following intra-cardiac electrical shocks using a proteomic approach in a sheep model

**DOI:** 10.1038/s41598-020-77346-x

**Published:** 2020-11-20

**Authors:** Alexandre Bodin, Valérie Labas, Arnaud Bisson, Ana-Paula Teixeira-Gomes, Hélène Blasco, Daniel Tomas, Lucie Combes-Soia, Paulo Marcelo, Elodie Miquelestorena-Standley, Christophe Baron, Denis Angoulvant, Dominique Babuty, Nicolas Clementy

**Affiliations:** 1 Service de Cardiologie, Centre Hospitalier Universitaire Trousseau Et EA7505, Faculté de Médecine, Université François Rabelais, Tours, France; 2 INRAE, CNRS, IFCE, UMR PRC, Université de Tours, 37380 Nouzilly, France; 3 INRAE, CHU de Tours, Plate-Forme de Chirurgie Et D’Imagerie Pour La Recherche Et L’Enseignement, Université de Tours, 37380 Nouzilly, France; 4 INRAE, ISP, Université de Tours, 37380 Nouzilly, France; 5 Imagerie Et Cerveau - UMR 1253, Université de Tours, Tours, France; 6 Plate-Forme ICAP, Centre Universitaire de Recherche en Santé, Université de Picardie Jules Verne, 80054 Amiens, France; 7 Transplantation, Immunologie et Inflammation T2i - EA 4245, Université de Tours, Tours, France

**Keywords:** Cardiac device therapy, Proteomic analysis

## Abstract

Implantable cardioverter-defibrillators (ICD) are meant to fight life-threatening ventricular arrhythmias and reduce overall mortality. Ironically, life-saving shocks themselves have been shown to be independently associated with an increased mortality. We sought to identify myocardial changes at the protein level immediately after ICD electrical shocks using a proteomic approach. ICD were surgically implanted in 10 individuals of a healthy male sheep model: a control group (N = 5) without any shock delivery and a shock group (N = 5) with the delivery of 5 consecutive shocks at 41 J. Myocardial tissue samples were collected at the right-ventricle apex near to the lead coil and at the right ventricle basal free wall region. Global quantitative proteomics experiments on myocardial tissue samples were performed using mass spectrometry techniques. Proteome was significantly modified after electrical shock and several mechanisms were associated: protein, DNA and membrane damages due to extreme physical conditions induced by ICD-shock but also due to regulated cell death; metabolic remodeling; oxidative stress; calcium dysregulation; inflammation and fibrosis. These proteome modifications were seen in myocardium both “near” and “far” from electrical shock region. N-term acetylated troponin C was an interesting tissular biomarker, significantly decreased after electrical shock in the “far” region (AUC: 0.93). Our data support an acute shock-induced myocardial tissue injury which might be involved in acute paradoxical deleterious effects such as heart failure and ventricular arrhythmias.

## Introduction

Sudden cardiac death (SCD) is defined as a “death from an unexpected circulatory arrest, usually due to a cardiac arrhythmia, occurring within an hour of symptoms onset, potentially reversed by medical intervention (e.g., defibrillation)”^1^. Implantable cardioverter-defibrillators (ICD) are meant to fight life-threatening ventricular arrhythmias and are associated with a reduction of all-cause mortality^2^. Ironically, electrical ICD-shocks (appropriate or inappropriate) have been shown to be independently associated with an increased mortality either in primary or secondary prevention^3–8^. The most common cause of death among patients who received any ICD shock was progressive heart failure.

Clinical evidences support the hypothesis of electrical shocks related cardiac tissue injury. A dose–response effect is indeed described: multiple shocks are more detrimental than one^6^ and the amount of shock energy is associated with all-cause death^9^. Moreover, modification of ICD programmation with shock reduction strategies are associated with a reduction of mortality^10–12^. Electrical shocks seem to be associated with myocardial injury as suggested by surrogates in humans: increase in biomarkers of myocardial injury during defibrillation testing (DFT) following ventricular fibrillation induction (cardiac troponin I, CK-MB, H-FABP)^13–17^ but also without any ventricular arrhythmias^18^, electrical changes during DFT (transient local injury current)^19^, and transient alteration of hemodynamics following DFT (duration and extent of the adverse effect were proportional to the shock strength)^20,21^.

However, little is known on specific mechanisms involved in acute shock-induced myocardial injuries. Proteomic approaches enable accurate characterization of proteome modifications in dynamic situations such as an acute stress, directly providing relevant information on altered biological cascades. Top-down and bottom-up proteomics strategies are the two main complementary approaches used^22^. Briefly, tissue profiling using MALDI-TOF MS allows in situ detection of intact peptides or proteins for qualitative and quantitative analyses. It is useful to detect degradation products and/or biomarkers within cellular and tissular environment^23^. This tissue profiling approach is then combined with TD HR-MS in order to identify finely the peptido- or proteoforms and to characterize their sequences and post-translational modifications (PTM). Bottom-up proteomics involve initial enzymatic protein digestions, and subsequent protein identifications from peptide mixtures^24^ before a quantification.

Our objective was to identify myocardial changes at the protein level immediately after ICD electrical shocks, using different qualitative and quantitative proteomic approches based on tissue profiling, top-down and bottom up strategies, on a sheep model.

## Materials and methods

Detailed methods are provided in supplemental Materials and Methods and have been used in previous studies^23,25^.

### Experimental settings and tissue collecting

Experimentations were performed on an adult sheep model (*Ovis aries*). Procedures were approved by an Ethical Committee protecting animal rights in France (*Comité d’éthique en expérimentation animale Val de Loire - CEEA VdL, Autorisation de Projet utilisant des Animaux à des Fins Scientifiques* #6118) and all experiments were performed in accordance with relevant guidelines and regulations. Implantation was performed under general anaesthesia induced by a Ketamine (Imalgene 10 mg/kg, Boehringer Ingelheim animal health, France) and Xylazine (Rompun 2% 0.05 mg/kg, Bayer healthcare, France) intravenous bolus injection. After loss of consciousness, the animal was intubated and the anaesthesia was maintained by inhalation of 3% isoflurane (Vetflurane, Virbac, France) carried by oxygen. In case of pain occurrence, an additional morphine bolus could be injected. The defibrillator implantation was carried out similarly to human implantation, using X-ray visualization. Through a left jugular approach using Seldinger technique, the defibrillator lead was positioned percutaneously at the right ventricular apex, screwed in the myocardium, and connected to the defibrillator box placed in a left lateral thoracic subcutaneous position.

Animals were distributed in 2 groups of 5 each. In the “electrical shock group”, 5 biphasic maximal energy shocks (i.e. 41 J, as routinely used in human^1^) were successively delivered, synchronized on R-wave to avoid any proarrhythmic effect. In the “control group”, no therapy was delivered after ICD lead positioning.

**Figure 1:**
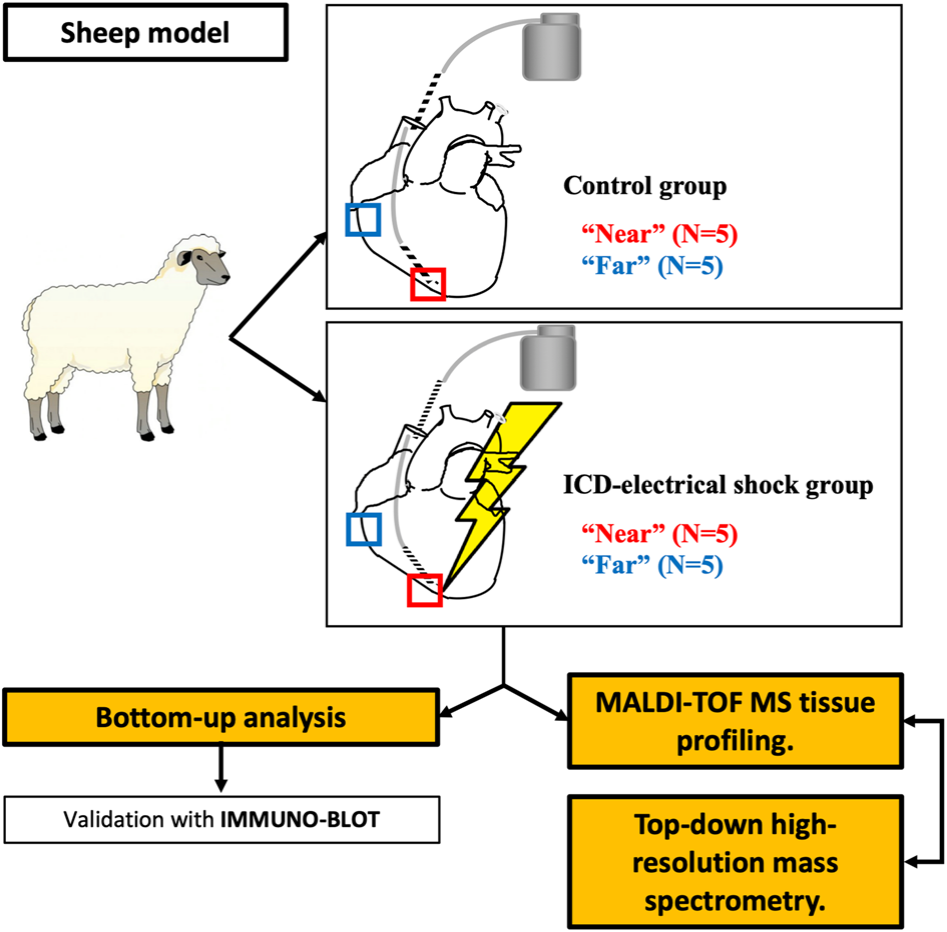
Experimental design. Five sheep in the “control group”: a defibrillation ventricular lead was placed at the right-ventricular apex and connected to a subcutaneous implantable cardiac defibrillator (ICD). No therapy was delivered nor any arrhythmia induced. Five sheep in the “electrical shock group”: ICD implantation was similar. 5 consecutive biphasic maximal energy shocks (41 J) were delivered without arrhythmia. Tissue samples were collected within 10 min at RV apex (“near”) and basal free wall (“far”). A MALDI-TOF MS tissue profiling coupled to a top-down high-resolution mass spectrometry was then performed in order to quantify and identify the intact proteins but also their post-translationnal modifications (PTM). A bottom-up analysis was also carried out, after enzymatic protein digestion, to identify and quantify proteins more exhaustively.

Animals were then sacrificed 5 min following last shock delivery (or lead positioning in the control group). Myocardial tissue samples were collected at the right-ventricle apex near to the lead coil (subgroup “near” from electrical shock region, N = 5 in the two groups) and at the right ventricle basal free wall region (subgroup “far” from electrical shock region, N = 5 in the two groups), and immediately snapped frozen in vapor of liquid nitrogen (Fig. 1).

### MALDI-TOF MS tissue profiling

Profiles of myocardial cryosections of each individuals were acquired using an UltrafleXtreme MALDI-TOF instrument (Bruker Daltonics, Bremen, Germany). For each cryosection, 20 average spectra were collected (resulting from a sum of 10,000 spectra).

For the comparative analysis of individuals peaks between control and electrical shock groups in the “near” and “far” regions, non-parametric Wilcoxon statistic test was used. Masses were considered statistically differential between groups if the *p* value was < 0.01 with a Fold Change (FC) ratio between the mean normalized intensity values > 1.5 or < 0.66. Receiver operating characteristic (ROC) curves were generated and masses with areas under the curve (AUC) > 0.8 were specially retained.

### Top-down high-resolution mass spectrometry

Extracted intact peptides/proteins were subjected to fractionation through chromatographic using two different chromatographic approaches (reversed-phase (RP) high-performance liquid chromatography (HPLC) and gel filtration (GF)). After separation and enrichment, each fraction was analyzed by on-line microflow liquid chromatography tandem high resolution mass spectrometry (µLC-MS/MS).

Proteo/peptidoform identification and structural characterization were performed using ProSight PC software v 4.0 (Thermo Fisher, San Jose). We applied a validation threshold with a E-Value of ≤ 1 × 10^−6^ (monisotopic) or ≤ 1 × 10^−8^ (average) presenting a C score > 3.

From the list of the idenfied proteo/peptidoforms by top-down MS, we annotated MALDI-TOF peak if the average mass [M + H]^+^ was within a ± 0.05% error mass tolerance.

### Bottom-up analysis

After protein extraction, samples from each individual (N = 5) of the 4 groups were pooled (electrical shock near and far regions, control group near and far regions with 5 samples for each group) and fractionated by SDS-PAGE. After staining with Coomassie Blue R-350, each lane was cut in 20 bands. Proteolytic digestion of gel pieces was carried out overnight with trypsin as described previously^25^.

All peptide mixtures were analysed by nanoLC-MS/MS using an Orbitrap Fusion mass spectrometer system (ThermoFisher Scientific, Bremen, Germany).

MS/MS ion searches were performed using Mascot search engine version 2.6 (Matrix Science, London, UK) via Proteome Discoverer 2.1 software (ThermoFisher Scientific, Bremen, Germany) against NCBIprot_mammals database.

For comparative analyses between control and electrical shock groups in the “near” and “far” regions, we employed Scaffold Q+ software (version 4.8.9, Proteome Software, Portland, USA) to apply two independent label-free quantitative methods (Normalized Weighed Spectra (NWS) and Extracted-ion chromatogram (XIC)). Significance was determined using *t* test where *p* < 0.05 was considered significant.

An immunoblot analysis was used to confirm our bottom-up quantification. Each individuals from each group in the far region were compared (i.e. 5 individuals from control group and 5 individuals from the electrical shock group). Samples from the bottom-up analysis extraction were used. To normalize the data, Ponceau S staining was used as previously described^26^.

## Results

### MALDI-TOF MS phenotyping of myocardial tissue

**Figure 2:**
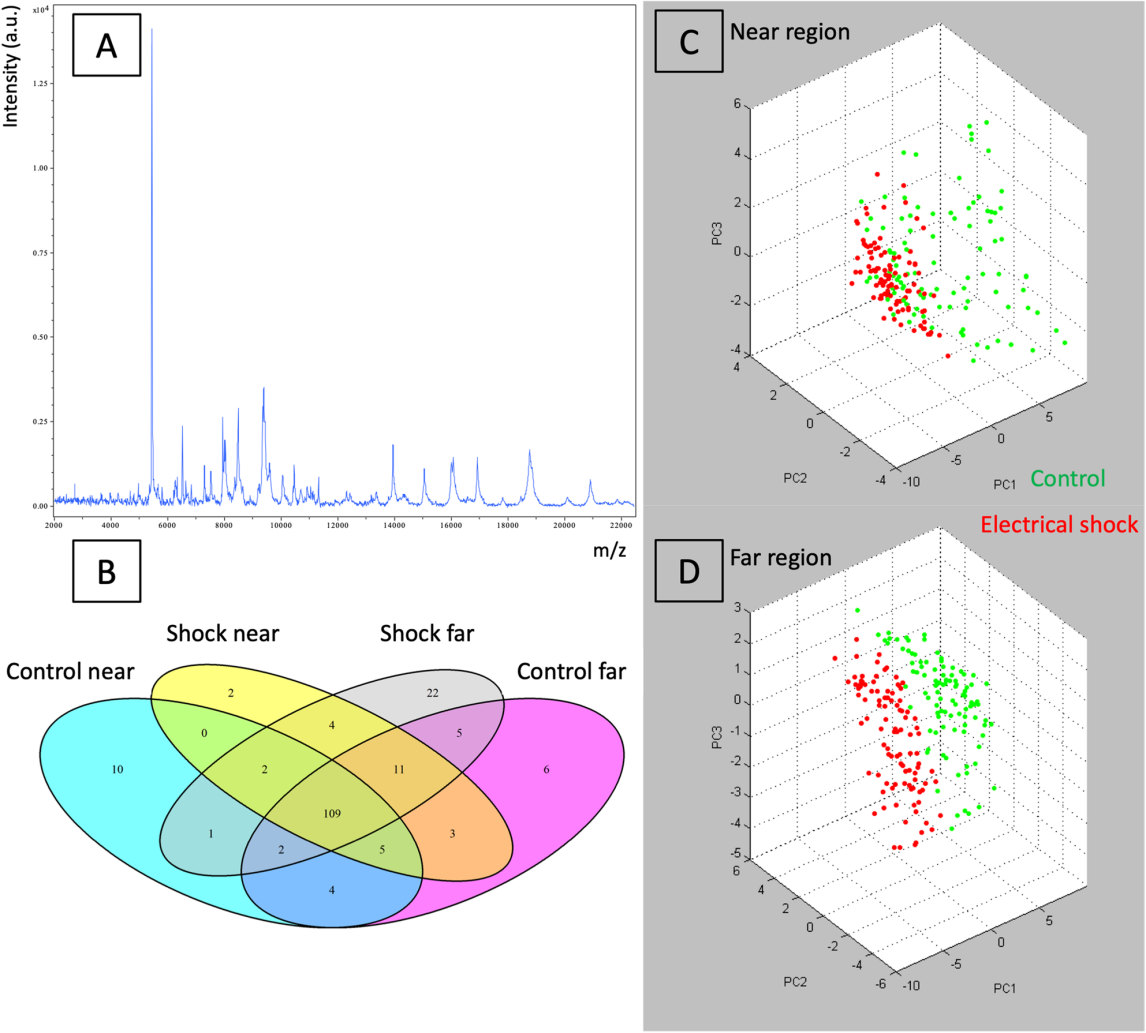
MALDI profiling dataset. Representative MALDI spectrum of a myocardial section in the “near” lead region after electrical shocks (**A**). Venn diagram with four main conditions (**B**). PCA analysis in “near” region (**C**) and in “far” region (**D**), electrical shock group (red dots) can be discriminate from control group (green dots). *PC* principal components, *a.u* arbitrary unit.* Images included have been created using ClinProTools 3.0 software (Bruker Daltonics, Bremen, Germany, *https://www.bruker.com/service/support-upgrades/software-downloads/mass-spectrometry.html*).*

A total of 100 mean spectra were collected for each region of the two groups. A representative spectrum is shown in Fig. 2A. A total of 133, 145, 136 and 156 m/z were detected in control “near”, control “far”, electrical shock “near” and electrical shock “far” groups respectively. A Venn diagram representing common and specific m/z in the four different conditions is also represented in Fig. 2B. The principal component analysis (PCA) performed with the different peaks was able to clearly discriminate the control and electrical shock groups in both regions (Fig. 2C,D).

Differential analysis between control and electrical shock in the “near” region characterized 32 m/z molecular species with a *p* value < 0.01 and a FC > 1.5 or < 0.66 including 20 m/z with an AUC > 0.8. In the “far” region, 34 m/z were different (*p* value < 0.01, FC > 1.5 or < 0.66), including 27 m/z with an AUC > 0.8 (Supplemental dataset 1).

### Top-down HR-MS proteomic analysis

#### Proteins identified by top-down HR-MS

We were able to identify 171 peptidoforms and proteoforms without redundancy, this represented 92 UniprotKB accession numbers corresponding to 73 unique genes. From this identification subset, we retained 68 peptidoforms and proteoforms automatically validated with high values of scoring with C score > 3 and E-values ranging from 9.06 × 10^–8^ to 2.35 × 10^–71^ in a mass range between 1211 and 18,459 Da (Supplemental dataset 2).

Among these, 8.82% corresponded to whole proteins, 13.24% to N-terminal fragments of original proteins, 26.47% to internal fragments and 51.47% to C-terminal fragments. Protein fragments were predominant and represented 91.17% of the validated identifications. The most cleaved proteins identified by TD were the myosin light chain II and the alpha-crystallin B chain.

To obtain an overview of the residues engaged in these cleavage sites, the occurrence of the amino acids at the end of the N-ter or internal fragments, and those above the internal or C-ter fragments, were noted. Top 5 most frequent aminoacids are represented in Table 1, the most frequent residues found at the end and above the fragment being A, D, F, G, L, R and S. These data suggest that the identified biomolecules are products of protease activities, from trypsin, chymotrypsin-like, caspase enzymes or other endopeptidases targeting specific sites and/or specific substrates. Using CASBAH and MEROPS databases, 2 peptidases were relevant for two identified peptidoforms: the matrix metallopeptidase 9 (MMP-9) which cleaves the alpha-crystallin B chain (*CRYAB*) in position 54^27^, and the mitochondrial processing peptidase beta-subunit (MPP-I) which cleaves the ATP synthase subunit delta (mitochondrial) in position 22^28^. These data suggest that the biomolecules fragments identified by TD are at least partially products of an enzymatic proteolytic activity.

**Table 1:** Occurrence of amino acid residues involved in protein cleavages generating N-terminal, C-terminal or internal fragments.

	Previous residues X-SEQUENCE	Terminal residues SEQUENCE-X
N-terminal fragment	–	ADGLR
Internal fragment	DFGLR	ADFLR
C-terminal fragment	ADFLS	–

The top five most frequent residues found at the end and above the 62 peptide fragments generated from original proteins are presented.

This subset of 68 identified peptidoforms and proteoforms matches to 22 unique genes with various interactions represented in a STRING network in supplemental Fig. 1. Considering all active interaction sources, including text mining, networks were mainly focused around sarcomere constitutants, protein metabolism (ubiquitin and heat-shock proteins) and mitochondrial energetic metabolism.

#### Annotation of tissue MALDI-TOF MS peaks to identify potential tissular markers of acute shock-induced myocardial tissue injury

Fourteen validated peptidoforms/proteoforms were assigned to m/z previously observed by MALDI-TOF profiling. This represented 14 UniprotKB accession numbers and 11 unique genes in a mass range between 2771 and 18,459 Da. Out of these biomolecules and considering the quantitative MALDI-TOF analysis, 3 differential m/z reached our predefined criteria in the “far” region (*p* value < 0.01, FC > 1.5 or < 0.66 and AUC > 0.8) (Table 2).

**Table 2:** MALDI-TOF tissular differential peaks identified by Top-down HR-MS.

MALDI-TOF differential peaks				Top-down HR-MS identification				
MALDI-TOF peak (m/z)	*p* value	Fold change (electrical shock/control)	AUC	Gene name	Protein name	BLAST (*ovis aries*)	E Value	C Score
**"Near" region**								
2770.86	< 0.000001	0.75	0.67	ALB	Serum albumin	Internal fragment, 24 amino-acids (aa)	4.7E-27	3.0
5105.57	0.00185	1.15	0.64	HSPB1	-	C-term fragment (56 aa), 100% identity: 27 kDa heat shock protein 1	9.06E-8	527.3
5443.32	0.00000232	1.22	0.7	-	-	C-term fragment (47 aa), 100% identity: cytochrome c oxidase subunit 7C. mitochondrial	1.97E-23	888.4
6219.54	< 0.000001	1.3	0.72	COX5A	Cytochrome c oxidase subunit 5A	Internal fragment (54 aa)	4.63E-42	609.8
6946.23	0.0026	1.16	0.62	HSP90AB1	-	N-term fragment (60 aa), 100% identity: heat shock protein HSP 90-beta	1.12E-41	1315.0
8188.31	0.00251	1.19	0.63	-	-	N-term fragment, 90.3% identity (56/62 aa): 26S proteasome complex subunit SEM1	2.55E-20	8.7
8491.39	< 0.000001	1.24	0.76	UBC	Ubiquitin C	N-term fragment (74 aa) with N-acetyl-l-methionine	3.44E-43	115.9
8568.22	< 0.000001	1.16	0.74	UBA52	Ubiquitin-60S ribosomal protein L40	N-term fragment (76 aa)	1.52E-62	150.2
10,847.84	0.00000675	1.15	0.69	HSP10	HSP10	Whole protein (101 aa) with *N*-acetyl-l-alanine	2.51E-18	167.6
16,925.88	< 0.000001	1.38	0.75	MB	Myoglobin	Whole protein (161 aa) with *N*-acetyl-l-methionine	1.04E-9	47.6
**"Far" region**								
2,771.39	0.00000262	1.24	0.69	ALB	Serum albumin	Internal fragment, 24 amino-acids (aa)	4.76E-27	3.0
5,105.58	< 0.000001	1.33	0.8	HSPB1	**-**	C-term fragment (56 aa), 100% identity: 27 kDa heat shock protein 1	9.06E-8	527.3
**5,443.3**	< 0.000001	**1.57**	**0.87**	**-**	**-**	**C-term fragment (47 aa), 100% identity: cytochrome c oxidase subunit 7C. mitochondrial**	**1.97E-23**	**888.4**
6,219.27	< 0.000001	1.26	0.77	COX5A	Cytochrome c oxidase subunit 5A	Internal fragment (54 aa)	4.63E-42	609.8
**6,947.27**	< 0.000001	**1.5**	**0.86**	**HSP90AB1**	**-**	**N-term fragment (60 aa), 100% identity: heat shock protein HSP 90-beta**	**1.12E-41**	**1315.0**
8,188.79	0.00000208	1.32	0.7	-	**-**	N-term fragment, 90.3% identity (56/62 aa): 26S proteasome complex subunit SEM1	2.55E-20	8.7
8,491.39	< 0.000001	1.25	0.89	UBC	Ubiquitin C	N-term fragment (74 aa) with *N*-acetyl-l-methionine	3.44E-43	115.9
8,565.03	< 0.000001	1.14	0.75	UBA52	Ubiquitin-60S ribosomal protein L40	N-term fragment (76 aa)	1.52E-62	150.2
10,849.29	< 0.000001	0.82	0.8	HSP10	HSP10	Whole protein (101 aa) with *N*-acetyl-l-alanine	2.51E-18	167.6
**18,458.61**	< 0.000001	**0.53**	**0.93**	**TNNC1**	**Troponin C**	**Whole protein (161 aa) with N-acetyl-l-methionine**	**2.07E-13**	**235.4**

In bold, 3 significantly differential m/z between electrical shocks and control groups in the MALDI-TOF phenotyping analysis (*p* value < 0.01, FC > 1.5, AUC > 0.8). Uncharacterized proteins were mapped to the corresponding *Ovis aries* orthologues by identifying the reciprocal-best-BLAST hits using blastp program (https://blast.ncbi.nlm.nih.gov/Blast.cgi). *AUC* area under the curve,* aa* amino-acids.

The m/z 5443 was assigned to the C-term fragment (47 aminoacids) of the subunit 7C of cytochrome-c oxidase, mitochondrial (*COX7C*), which was more abundant after electrical shocks in the “far” region (*p* value < 0.000001, FC = 1.57, AUC = 0.87). No known specific caspase or peptidase was found.

The m/z 6947 was annotated as the N-term fragment (60 aminoacids with initiator methionine removal) of the heatshock protein 90-beta (*HSP90AB1*), which was more present after electrical shocks in the “far” region (*p* value < 0.000001, FC = 1.5, AUC = 0.86). This initial methionine cleavage has not been previously described. This fragment finishing with a D residue, known as a potential targeted site of caspase-mediated proteolysis during apoptosis, suggested a caspase proteolysis. Indeed, HSP90-beta is known as a substrate of caspases^29^.

The m/z 18,458 was annotated as the troponin c *(TNNC1),* corresponding to the whole protein (161 aminoacids) with an acetylation on N-terminal methionine and was less abundant after electrical shocks in the “far” region (*p* value < 0.000001, FC = 0.53, AUC = 0.93).

### Bottom-up proteomic analysis

All data are presented and summarized in subsections “final table” for each region in the supplemental dataset 3.

#### Global proteome identified in “near” and “far” regions in control and electrical shock groups

**Figure 3:**
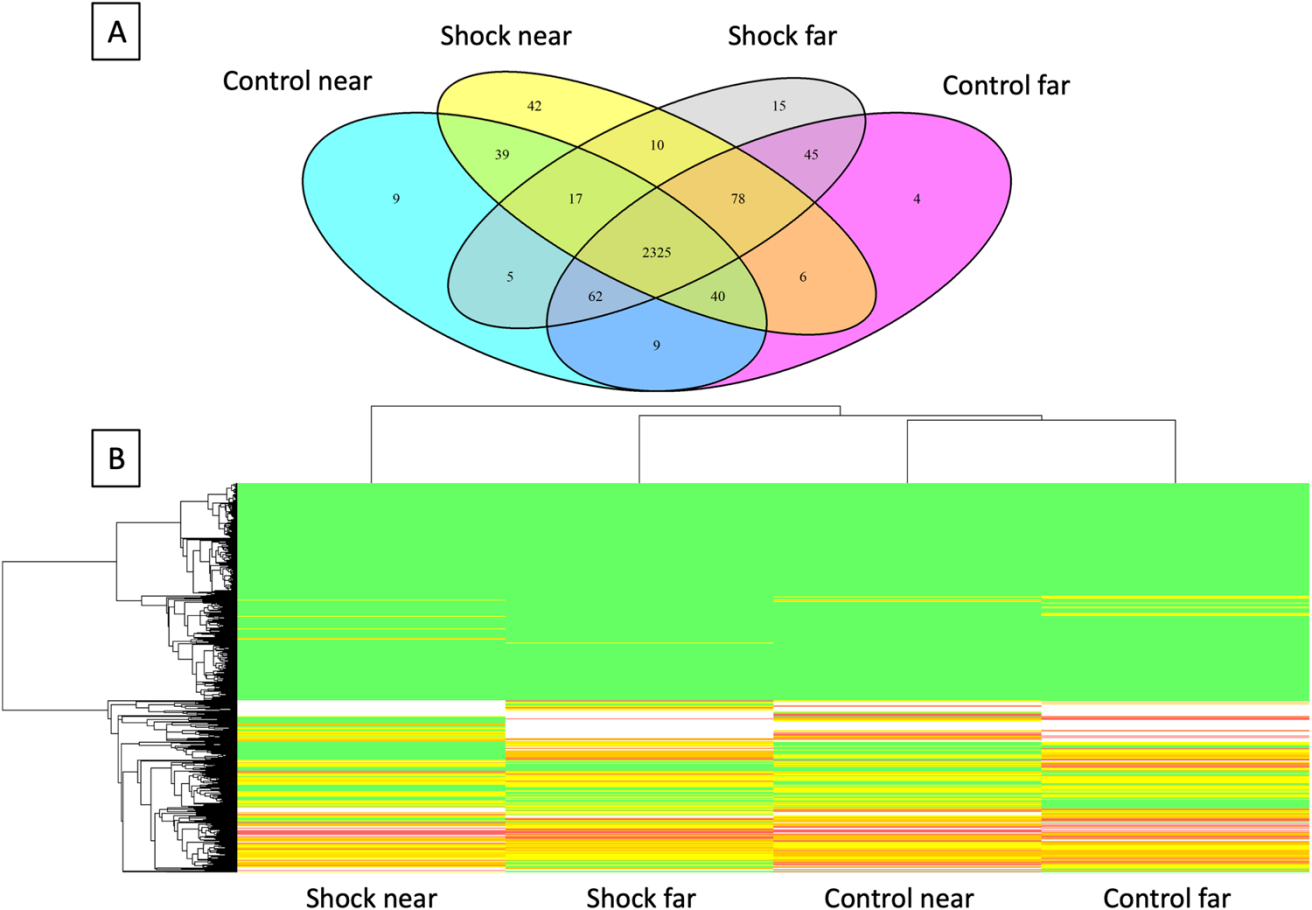
Bottom-up quantitative proteomic dataset of all proteins. (**A**) Venn diagram with shared and specific identified proteins according to each conditions. (**B**) Heat map of all identified proteins in two groups with the bottom-up analysis. *Green: high abundance, yellow/red: low abundance, white: very low abundance and absence. Images included have been created using Scaffold software (v 4.8.9, Proteome Software, Portland, USA, *https://www.proteomesoftware.com/products/scaffold/*).*

A total of 1257 clusters corresponding to 2706 proteins were identified. A Venn diagram is represented in Fig. 3A showing the common and specific identified proteins in each condition. A heat-map of differential proteins (*p* < 0.01) in 4 conditions is also represented (Fig. 3B). Control groups were the closest groups, and electrical shock “near” group had the highest changes.

#### Proteins identified in the “near” region

**Figure 4:**
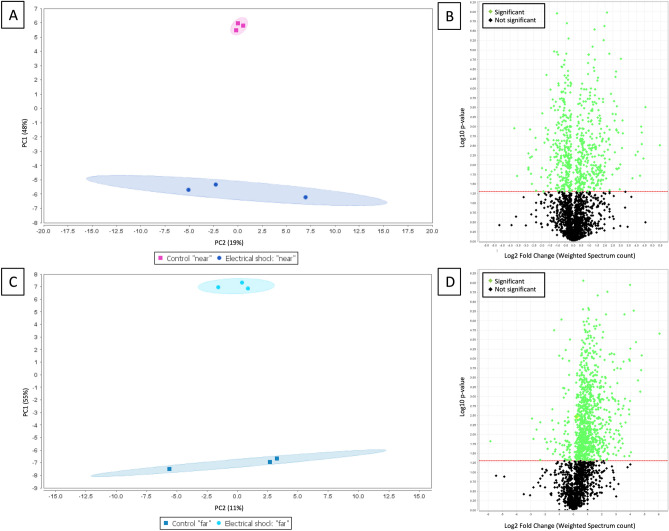
Bottom-up quantitative proteomic dataset of differential proteins in each region. (**A**) Principal components analyses of protein abundances in control and electrical shocks groups across the “near” region. (**B**) Volcano plot, providing a visual representation of differential protein regulation between electrical shocks and control groups in the “near” region. Green dots represent significantly regulated (*p* < 0.05) proteins identified between the two groups. (**C**) PCA across the “far” region. (**D**) Volcano plot in the “far” region. *PC* principal components.* Images included have been created using Scaffold software (v 4.8.9, Proteome Software, Portland, USA, *https://www.proteomesoftware.com/products/scaffold/*).*

In the “near” region, 1055 clusters corresponding to 2225 proteins were identified. Electrical shock and control groups were well discriminated with the PCA analysis (Fig. 4A). Significantly differential proteins seemed harmoniously increased and decreased after electrical shocks as seen on the volcano plot (Fig. 4B).

A total of 1775 NCBI reference sequences had an average normalized weighted spectrum (NWS) ≥ 5: 1104 were differential with a *p* value < 0.05 in either peptide-pattern based on XIC or NWS quantification method, and 513 with a *p* value < 0.05 in both methods. After cluster sorting, gene redundancy suppression, elimination of keratins and elimination of redundant proteins, proteins from 48 unique genes were less abundant and proteins from 38 unique genes more abundant after electrical shocks (*p* value < 0.05 in NWS or XIC method associated to a fold change/ratio ≤ 0.5 or ≥ 2) (Supplemental dataset 3).

The main decreased proteins after electrical shocks were cytoskeleton and sarcomere constituants (including the tropomyosin family), oxygen transport or stockage proteins and actors of protein metabolism including heatshock proteins. Proteins involved in gluconeogenesis, fatty acid beta-oxidation, respiratory chain, metabolism of reactive species and in calcium regulation (calsequestrin-2, sarcoplasmic reticulum histidine-rich calcium-binding protein) were also less abundant. More specific proteins were as well decreased such as cAMP-dependent protein kinase type I-alpha regulatory subunit and polymerase I and transcript release factor. These proteins have various interactions as shown in the STRING network representation (Supplemental Fig. 2A). Considering all active interaction sources, including text mining, networks of decreased proteins were mainly focused around sarcomere constituants, oxygen transport and stockage, and mitochondrial energetic metabolism.

Main increased proteins after electrical shocks were involved in glycolysis, fatty acid metabolism, respiratory chain, detoxication of reactive species and in protein metabolism including heat shock proteins. More specific proteins were also more abundant after electrical shocks such as protein S100-A9 and filamin-A. Proteins from the extracellular matrix (integrin), from the desmosome (desmoplakin and plakoglobin) and proteins involved in fibrosis (galectin 3-like, galectin-7, transglutaminase-2) were also more abundant after electrical shocks. Two proteins appeared after electrical shocks and were specific: aldehyde oxidase involved inoxidative stress and serpin peptidase inhibitor, clade A, member 1 involved in inflamamtion. These proteins have also various interactions (Supplemental Fig. 2B). Considering all active interaction sources, including text mining, networks of increased proteins were mainly focused around glycolysis and proteasome constituants.

#### Proteins identified in the “far” region

In the “far” region, 1150 clusters corresponding to 2254 proteins were identified. Electrical shocks and control groups were well discriminated with the PCA analysis (Fig. 4C). Significantly differential proteins were mainly increased after electrical shocks as seen on the volcano plot (Fig. 4D).

A total of 1743 NCBI reference sequences had an average NWS ≥ 5: 1310 were differential with a *p* value < 0.05 in either XIC or NWS method, and 544 with a *p* value < 0.05 in both methods. After cluster sorting, gene redundancy suppression, elimination of keratins and elimination of redundant proteins, proteins from 35 unique genes were less abundant after electrical shocks, and proteins from 116 unique genes were more abundant (*p* value < 0.05 in NWS or XIC method associated to a fold change/ratio ≤ 0.5 or ≥ 2) (Supplemental dataset 3).

Main decreased proteins in “far” region after electrical shocks were cytoskeleton and sarcomere constituants and proteins involved in oxygen transport and stockage. Proteins involved in fatty acid metabolism, respiratory chain, detoxication of reactive species and in protein metabolism were also less abundant. These proteins have various interactions as shown in the STRING network (Supplemental Fig. 3A). Considering all active interaction sources, including text mining, networks of decreased proteins were mainly focused around sarcomere constituants and proteins for oxygen transport and stockage.

Increased proteins after electrical shocks were numerous and mainly involved in glycolysis, gluconeogenesis, fatty acid metabolism, citric acid cycle, respiratory chain, detoxication of reactive species and protein metabolism as well as constituants of the cystokeleton and sarcomere. Proteins involved in calcium regulation (sarcoplasmic reticulum histidine-rich calcium-binding protein and protein phosphatase 1) and regulated cell death but also in cell repair were more abundant after electrical shocks. PGC-1 and ERR-induced regulator in muscle protein 1, cAMP-dependent protein kinase catalytic subunit beta (PKA C-beta), CRP and immunoglobulins were also more abundant after electrical shocks. These proteins have many interactions as shown in Supplemental Fig. 3B. Considering all active interaction sources, including text mining, networks of increased proteins were numerous and mainly focused around mitochondrial energetic metabolism, protein metabolism and cytoskeleton/sarcomere constituants.

Bottom-up quantitative analysis was validated with an immunoblot analysis (Supplemental dataset 4).

## Discussion

Our study is the first global proteomic approach that investigates acute myocardial physiopathological impact of electrical shocks in a large animal model.

### Cytolysis

Many biomolecules identified by TD were fragments which were, at least partially, products of an enzymatic proteolytic activity (residues engaged in these cleavage sites, MMP-9 and MMP-I). Proteasome constituants were more abundant in the bottom-up analysis in both regions following electrical shocks. Ubiquitin family proteins were increased, suggesting a degradation of damaged proteins by electrical shocks via proteasome after ubiquitin tagging^30^. This protein denaturation may have been detected by stress proteins involved in cellular stress response like heatshock proteins (HSP). Our results suggest a significant decrease of HSP’s chaperone effect in both regions following electrical shocks.

Extreme physical conditions such as high temperature induced by the high electrical gradient during ICD shock may also cause non enzymatic cell degradation. Proteins such as RAD23 play a keyrole in the nucleotide excision/repair pathway, especially in the recognition of thermic DNA damages^31^. ATP-dependent (S)-NAD(P)H-hydrate dehydratase is also known to be transcripted after osmotic or heat stress conditions, in order to convert the abnormal metabolite NAD(P)HX to NAD(P)H^32^. Cell membrane also seemed to be damaged as Polymerase I and transcript release factor (PTRF1), an indispensable components of the membrane repair machinery^33^, was increased after electrical shocks. Membrane disruption was previously described with electroporation injury in high gradient area^34^, but this effect might extend further than the myocardium just surrounding the defibrillating coil.

Similarly, a decrease in the major constituants of the cytoskeleton and the sarcomere, likely due to protein degradation, was also observed.

Beyond direct myocardial injury, we show that regulated cell death may also play a major role in cardiac dysfunction following electrical shocks. Decrease in inositol monophosphatase^35^, and increase in markers of intrinsic apoptosis (VDAC1*,* VDAC2, HINT2*)*, or parthanatos (poly [ADP-ribose] polymerase 6, AIFM1*)*^36^ were observed. Calreticulin may also be implied^37^. This is concordant with indirect evidence of apoptosis in human^17^.

To summarize our findings, electrical shocks induce DNA, proteins and membranes damages, both directly through extreme physical conditions, and through an enzymatic proteolytic activity including caspases of regulated cell death, into right ventricular myocardium.

### Metabolic remodeling and oxidative stress

Up to 41% of differential identified proteins through the bottom-up play a major role in energy metabolism. Increase of glycolytic pathway was associated with a decrease of upstream keystep of fatty acid metabolism (ACADVL) and gluconeogenesis. This switch from fatty acid β-oxidation to glycolysis is involved in heart failure (HF)^38^. This deep modification of energy metabolism induced by electrical shocks and such metabolic shifts might participate to cardiac dysfunction^39^.

Decrease in hemoglobin and myoglobin after electrical shock may also lead to a limitation of oxygen.

The abundance of filamin-A, involved in mitochondrial hyperfission induced by hypoxia following myocardial infarction, by interacting with Dynamin-related protein 1, may participate to the mitochondrial ultrastructural alterations in this region already described in dogs^40^, and thus metabolic modifications^41^.

Aldehyde oxidase production also shows a generation of reactive oxygen species (ROS) such as hydrogen peroxide. Increase in several proteins involved in detoxication of oxygen/nitrogen reactive species suggest an adaptive response to increased oxidative stress. This is concordant with some available data suggesting free-radical generation in dogs following transthoracic shocks^42^.

Finally, increase in peroxisome proliferator-activated receptor γ coactivator 1 (PGC-1) and estrogen-related receptor (ERR)-induced regulator in muscle protein 1 (Perm1), may participate to the regulation of muscle-specific transcriptional programs, such as mitochondrial biogenesis and oxidative metabolism^43^.

### Calcium regulation

In a rat model, the decrease in calsequestrin-2 (CASQ2) observed in our study was associated with a shorter calcium-release phase, but an accelerated restitution of calcium-release sites, with subsequent proarrhythmic calcium concentrations oscillations triggering delayed after depolarizations (DADs)^44^. Excessive diastolic calcium release may also play a role in the development of HF^45^. Histidine rich calcium binding protein (HRC) overexpression may also lead to a depressed contractility^46^.

Protein phosphatase 1 (PP1) is also central in SERCA2a regulatome as it dephosphorylates the phospholamban (PLN)^47^. An increase of PP1R7 may decrease PP1 activity, dephosphorylated PLN concentration, and thus increase SERCA2a activity and intrasarcoplasmic calcium handling.

These mechanisms of alteration of calcium regulation, as previously described in Langendorff rats’ hearts^48^, may be implied in HF and ventricular arrhythmias following electrical shocks.

### Inflammation and fibrosis

Serpin peptidase inhibitor, clade A, member 1 (also called alpha-1 antitrypsin) and protein S100-A9 increase following electrical shocks, along with immunoglobulins and CRP, suggest an associated inflammatory response following electrical shocks in a large surrounding area, which may participate to cardiac dysfunction.

The acute increase in galectin 3-like, galectin-7 and transglutaminase-2 (TGM2) we observed following electrical shocks in near region, might lead to the local progressive fibrotic invasion described in chronic studies^49^.

### N-acetylated troponin C as a tissular biomarker?

Acetylated lysine on troponin C was decreased after electrical shock. This acetylation, particularly on the N-terminal part, could modulate troponin C structural stability and interaction with calcium in rabbits^50^. Its decrease has also been described in skeletal muscle amyotrophy in rats^51^. The impressive AUC of 0.93 found in our study, might make N-acetylated troponin C a useful tissular biomarker to compare electrical shock tissue injury with different (extracardiac) defibrillation techniques. Further mechanistical investigations and validations are needed.

### Near and far regions

Modifications were observed in both regions. However, more changes were seen in the far region. In the bottom-up, significantly differential proteins seemed harmoniously increased and decreased after electrical shocks in near region when they were mainly increased in the far region. In our opinion, and moreover with the cytolisis previously described, electrical shock induced local destruction without specific pathways near the electrical shock area and more specific mechanisms, possibly compensatory, far from the shock. This would also explain why the top-down analysis mainly identified biomarkers in the far region.

### Limitations

Samples were collected within minutes following electrical shocks. In this relatively short timing, some mechanisms of the cellular machinery may have not been fully involved. However, the effects of shocks seem to be maximal in the early time after shock, especially regarding hemodynamics alteration^21^ and proarrhythmic effects, explaining our choice of collecting timing. Moreover, eukaryote proteins through the polyribosome can be synthesized in about 10 s for the smallest ones^52^. Evaluation of functional changes at later time points may be interesting in further studies, especially to observe the potential reversibility of described mechanisms and their involvement in chronic heart failure development.

A healthy adult sheep model was used, while ICDs are mainly implanted in HF patients with diseased myocardium (ischemic heart disease, dilated cardiomyopathy, hypertrophic cardiomyopathy…^1^). However, pure intrinsic effects of electrical shocks are better apprehended on a healthy myocardium without possible interacting modifications of proteome by different stages of HF or different pathological animal models. Our study also shows the potential deleterious effects in patients without patent cardiomyopathy, such as patients with channelopathies for instance. Further analyses on an HF animal model may be needed.

## Conclusions

Proteomic approaches enable accurate characterization of proteome modifications in dynamic situation such as electrical shocks. Several potential mechanisms involved in shock related cardiac tissue injury were identified: direct cellular damages due to extreme physical conditions, regulated cell death, metabolic remodeling and oxidative stress, calcium dysregulation, inflammation and fibrosis. These mechanisms had an extent towards areas outside the myocardium just surrounding the defibrillation coil.

Our data support an acute shock-induced myocardial tissue injury which might be involved in acute paradoxical deleterious effects such as heart failure and ventricular arrhythmias.

## Supplementary information

Supplementary Information.

Supplementary Dataset 1.

Supplementary Dataset 2.

Supplementary Dataset 3.

Supplementary Dataset 4.

Supplementary Figure 1.

Supplementary Figure 2.

Supplementary Figure 3.

Supplementary Legends.

## Abbreviations

ACN: Acetonitrile
AUC: Areas under the curve
CV: Coefficient of variation
DFT: Defibrillation threshold testing
FA: Formic acid
FC: Fold change
GeLC-MS/MS: SDS-PAGE and in-gel trypsin digestion of proteins combined to peptides analysis by nanoliquid chromatography coupled to high resolution tandem mass spectrometry
GF: Gel filtration
HCCA: Alpha-cyano-4-hydroxycinnamic acid
HF: Heart failure
HPLC: High-performance liquid chromatography
ICD: Implantable cardiac defibrillator
ITO: Indium tin oxide
MALDI-TOF MS: Matrix-assisted laser desorption/ionization time-of-flight mass spectrometry
NWS: Normalized weighed spectra
PCA: Principal component analyses
PTM: Post-translational modification
RP: Reversed-phase
RV: Right ventricle
SCD: Sudden cardiac death
SDS–PAGE: Sodium dodecyl sulfate–polyacrylamide gel electrophoresis
TD HR-MS: Top-down high-resolution mass spectrometry
TFA: Trifluoroacetic acid
XIC: Extracted-ion chromatogram
µLC-MS/MS: Microflow liquid chromatography coupled to tandem mass spectrometry
