# Supplementary material for: Acute pathophysiological myocardial changes following intra-cardiac electrical shocks using a proteomic approach in a sheep model
Source: Sci Rep. 2020 Nov 20;10:20252. doi: 10.1038/s41598-020-77346-x (PMC7679418; doi:10.1038/s41598-020-77346-x)
Supplement: Supplementary file 1 — Supplementary Information. [file 41598_2020_77346_MOESM1_ESM.docx]

**Acute pathophysiological myocardial changes following intra-cardiac electrical shocks using a proteomic approach in a sheep model.**

Alexandre BODIN*, MD^1^, Valérie LABAS^2-3^, Arnaud BISSON, MD^1^, Ana-Paula TEIXEIRA-GOMES^4^, Hélène BLASCO, PhD^5^, Daniel TOMAS^2-3^, Lucie COMBES-SOIA^2-3^, Paulo MARCELO^6^, Elodie MIQUELESTORENA-STANDLEY, MD^7^, Christophe BARON, MD, PhD^7^, Denis ANGOULVANT, MD, PhD^1,7^, Dominique BABUTY, MD, PhD^1^, and Nicolas CLEMENTY, MD^1,7^.

^1^ Service de Cardiologie, Centre Hospitalier Universitaire Trousseau et EA7505, Faculté de Médecine, Université François Rabelais, Tours, France

^2^ INRAE, CNRS, Université de Tours, IFCE, UMR PRC, 37380 Nouzilly, France

^3^ INRAE, Université de Tours, CHU de Tours, Plate-forme de Chirurgie et d’Imagerie pour la Recherche et l’Enseignement, 37380 Nouzilly, France

^4^ INRAE, Université de Tours, ISP, F-37380, Nouzilly, France

^5^ Imagerie et Cerveau - UMR 1253, Université de Tours, Tours, France

^6^ Plate-forme ICAP, Centre Universitaire de Recherche en Santé, Université de Picardie Jules Verne, 80054 Amiens, France

^7^ Transplantation, Immunologie et Inflammation T2i - EA 4245, Université de Tours, Tours, France

**SUPPLEMENTAL MATERIALS AND METHODS**

**Experimental settings and tissue collecting**

Sheep model (*Ovis aries*) was chosen for its similarities to the human heart regarding size and anatomy. It was preferred to a pig model, in whom amiodarone infusion during implantation to avoid ventricular arrhythmia is needed. Dog model also presents major ethical limitations. All sheep were males to avoid hormonal disturbances, most of implanted human patients being males; developmental stage was 5-year old adult. Median weight of sheep was 80.5kg (interquartile range: 72-84.75kg).

The implanted device was a defibrillator model Incepta F162 (Boston Scientific, Marlborough, MA, USA), designed for human use, with a maximum energy delivery of 41 joules (equivalent to approximatively 850 volts). A 9F single-coil DF-4 defibrillator lead, model 6935M (Medtronic, Minneapolis, MN, USA), was used for intra-cardiac implantation.

Implantation was performed under general anaesthesia induced by a Ketamine (Imalgene 10mg/kg, Boehringer Ingelheim animal health France) and Xylazine (Rompun 2% 0.05mg/kg, Bayer healthcare) intravenous bolus injection. After loss of consciousness, the animal was intubated and the anaesthesia was maintained by inhalation of 3% isoflurane (Vetflurane, Virbac France) carried by oxygen. In case of pain occurrence, an additional morphine bolus could be injected. The procedure was then carried out similarly to human implantation, using X-ray visualization (Arcadis Avantic, Siemens), with an overall procedure duration of approximatively 1 hour. Through a left jugular approach using Seldinger technique, the lead was positioned percutaneously at the right ventricular apex, screwed in the myocardium, and connected to the defibrillator box placed in a left lateral thoracic subcutaneous position. All procedures were performed by a single experienced operator.

In the “electrical shock group”, 5 cathodal biphasic shocks at maximal energy (41 J) were successively delivered, over a period of approximatively 90 seconds, synchronized on R-wave to avoid any proarrhythmic effect. In the “control group”, right ventricular lead was similarly placed at the right ventricular apex, without any delivered therapy or induced arrhythmia.

Animal was sacrificed within 5 minutes after the last electrical shock (shock group) or lead positioning (control group) (Dolethal 15mL bolus, Vetoquinol France), and heart was immediately explanted. Myocardial tissue samples (approximately 2x2x1cm) were collected at the right-ventricle apex near to the lead coil (subgroup “near” from electrical shock region, one sample per animal, i.e N=5 in the two groups) and at the right ventricle basal free wall region (subgroup “far” from electrical shock region, one sample per animal, i.e N=5 in the two groups) (**Figure 1**). Whole tissue samples were snapped frozen in vapor of liquid nitrogen within 10 minutes after last electrical shock or lead positionning and kept at -80°C until use.

**Chemicals**

All chemicals were provided by Sigma-Aldrich (Saint-Quentin Fallavier, France) unless others stated. Alpha-cyano-4-hydroxycinnamic acid (HCCA) from Sigma-Aldrich was house recrystallized.

**MALDI-TOF MS tissue profiling**

Similar methods were previously used^1^. The organ organ was placed at -20°C for 1h in the microstat chamber before tissue sections. Myocardial sections were cut using a Cryo-Star HM 560 cryostat (Microm, Francheville, France) with a specimen holder chilled at −20°C. The 12-µm thick sections were thaw mounted onto conductive Indium Tin Oxide (ITO)-coated microscopic slides (Bruker Daltonics, Wissembourg, France). The slides were placed in a vacuum dessicator for 1h. An optimal delipidation was obtained using three 30-seconds baths of 95% ethanol. The slides were then placed in a vacuum dessicator for 1h.

Myocardial sections were scanned before and after matrix deposition using a histology slide scanner (Opticlab H850 scanner, Plustek). Matrix solution was freshly prepared by saturating the solid HCCA crystals in 1mL of 50% acetonitrile (ACN)/50% H20 acidified by 0.2% trifluoroacetic acid reagent (TFA). For each sample, the matrix (0.5 µL x 3) was spotted manually homogenously on the myocardial sections using the dried droplet method. External calibrant was deposed near the tissue section using a mixture of peptides and proteins (1 µL of HCCA matrix plus 1 µL of calibrant solution containing Glu1-fibrinopeptide B, ACTH (fragments 18–39), insulin and ubiquitin, both at 1 pmol/μL, 2 pmol/μL cytochrome C, 4 pmol/μL myoglobin and 8 pmol/μL trypsinogen. The matrix was allowed to evaporate slowly at room temperature for 30 min, before MALDI-TOF analysis.

UltrafleXtreme MALDI-TOF instrument (Bruker Daltonics, Bremen, Germany) was equipped with a Smartbeam laser (Nd:YAG, 355 nm) at 2 kHz laser repetition rate that was controlled by FlexControl 3.0 software (Bruker Daltonics, Bremen, Germany). Spectra were obtained in positive linear ion mode in the m/z 1,000–30,000 range. The accelerating voltage was set to 25 kV. Anatomical regions of interest (ROIs) were manually defined using the histological image by targeting each matrix spots with a probe spot diameter of 1,500 µm using FlexImaging 4.0 software (Bruker Daltonics, Bremen, Germany). For each spot, an average spectrum was collected resulting from a sum of 500 consecutive laser shots, in 20 random walk shot steps (10,000 spectra). For acquisitions, analyses were piloted using FlexImaging 4.0 and FlexControl 3.0 softwares. To increase mass accuracy (mass error tolerance < 0.05%), internal calibration was achieved to all spectra using FlexAnalysis software by performing a lock mass correction on the most abundant mass (5,443.2 m/z). Similar setup were previously used^2,1^.

Spectra were integrated in ClinProTools 3.0 software (Bruker Daltonics, Bremen, Germany). Data analysis, as previously described^3^, began with an automated raw data pre-treatment workflow, comprising baseline subtraction (Top Hat, 10% minimum baseline width), two smoothing using the Savitzky-Golay algorithm. Spectra realignment was performed using prominent peaks (maximal peak shift 2000 ppm, 30% of peaks matching most prominent peaks, exclusion of spectra that could not be recalibrated). Normalization on peak intensity was performed using the Total Ionic Count (TIC) in order to display and compare all spectra on the same scale. Automatic peak detection was applied to the total average spectrum with a signal/background noise greater than 2. All spectra were processed using the same parameters. 31.3±3.7 mean spectra were obained for each organ and were manually filtered in order to obtain 20 spectra for each organ. The reproducibility for tissue analysis linked directly to the biological and individual variability in addition to the spectrometer process (technical variability) was evaluated by a coefficient of variation (CV) on the 5 control and 5 electrical shock samples for each region (“near” and “far”). Mean technical CV values did not exceed 28% and 27% for “near” and “far” regions, respectively.

**Top-down high-resolution mass spectrometry**

*Fractionation with GF and RP-HPLC*

The sample with the highest number of peaks from the electrical shock “near” group in the profiling analysis was chosen.

Protein extraction was performed by sonication in 600 µl of 6 M Urea 50 mM Tris-HCl pH 8.8 buffer containing protease inhibitor mixture. Sample was centrifuged during 45 min at 13,000 rpm and 4°C. One milligram of the extracted intact peptides/proteins contained in supernatants were subjected to fractionation through chromatographic separation on an UltiMate 3000 RSLC system controlled by Chromeleon version 6.80 SR13 software (Thermo Scientific Dionex) using two different chromatographic approaches, as previously described^1,4^.

First separation method was based on the biomolecules separation by hydrophobicity with reversed-phase (RP) high-performance liquid chromatography (HPLC) using an XBridge BEH C18 column (250 × 4.6 mm i.d., particule size 5 μm; Waters, Guyancourt, France). The column temperature was maintained at 30°C. Mobile phases for the chromatographic separation consisted of (A) 0.1% (v/v) trifluoroacetic acid (TFA) in water, and (B) 0.1% (v/v) TFA in acetonitrile. The gradient elution was carried out at a flow rate of 1 mL/min with 10% B for 5 min, 60% B at 45 min, 95% B at 47 min, constant 95% B for 2 min and then back to 10 % B at 51 min. A second approach was based on the biomolecules separation by molecular weight using a Superdex 75 10/300 GL gel filtration (GF) column (GE Healthcare Life Sciences) in 100 mM ammonium bicarbonate buffer.

Thus, 45 and 42 fractions were generated by RP and GF liquid chromatography processes, respectively. Samples from the RP were immediately vacuum-dried and kept at −20 °C until further analyses. All fractions from the GF were, after a vacuum-drying, desalted and enriched a second time using ZipTip C4 SPE (Millipore, Saint-Quentin-en-Yvelines, France) and eluted with a solution of 50% ACN in presence of formic acid 1% (FA). Samples were immediately vacuum-dried and kept at −20 °C until further analyses.

*MALDI-TOF mass fingerprint of fractions*

Each generated fraction was analyzed by MALDI-TOF to detect enriched molecular species. Matrix solution was freshly prepared by saturating the solid HCCA crystals in 1mL of 50%ACN/50%H20 acidified by 0.1%TFA. 1 µL was spotted (in triplicate) onto a Bruker 384-position polished steel sample plate and overlayed with 1 µL of matrix solution. The sample and the matrix (1:1, v/v) were loaded on the target using the dried droplet method. Profiles were acquired using the UltrafleXtreme MALDI-TOF instrument (Bruker Daltonics, Bremen, Germany). Spectra were obtained in positive linear ion mode in the m/z 1,000–30,000 range. For each spot, a mean spectrum was collected resulting from a sum of 500 consecutive laser shots, in 4 random walk shot steps (2,000 spectra). External calibration was followed using a mixture of peptides and proteins (1 µL of HCCA matrix plus 1 µL of calibrant solution as previously described).

Spectra were integrated in ClinProTools 3.0 software (Bruker Daltonics, Bremen, Germany). Data analysis, as previously described^3^, began with an automated raw data pre-treatment workflow, comprising baseline subtraction (Top Hat, 10% minimum baseline width), two smoothing using the Savitzky-Golay algorithm. All spectra were processed using the same parameters without alignment. Normalization on peak intensity was performed using the Total Ionic Count (TIC) and automatic peak detection was applied to the total average spectrum with a signal/background noise greater than 2. All mass fingerprints were visualized using gel view representation and all observed masses were extracted using FlexAnalysis.

*MicroLC-MS/MS and peptides/proteins identification*

Each HPLC fractions was analyzed by on-line microflow liquid chromatography tandem mass spectrometry (µLC-MS/MS). Furthermore, fractions with mass of interest of the tissue profiling identified in the previous step were pooled and re-analyzed in order to improve the probability of identification results. All experiments were performed on a dual linear ion trap Fourier Transform Mass Spectrometer (FT-MS) LTQ Orbitrap Velos Pro (Thermo Fisher Scientific, Bremen, Germany) coupled to an Ultimate 3000 RSLC Ultra High-Pressure Liquid Chromatographer (Dionex, Amsterdam, The Netherlands) controlled by Chromeleon Software (version 6.8 SR11; Dionex, Amsterdam, The Netherlands).

Similar methods were previously used^1^. Ten microliters of each sample were injected using µL-pickup mode and loaded on a trap column (Monolithic PS-DVB PepSwift, 200 µm inner diameter x 5 mm long). Mobile phases consisted of (A) 0.1% formic acid, 95.9 % water, 4 % acetonitrile (v/v/v) and (B) 0.1% formic acid, 15.9 % water, 84% acetonitrile (v/v/v). Biomolecules were automatically preconcentrated for 10 min at 10µL/min with 4% solvent B. The separation was conducted using a Dionex column (Monolithic PS-DVB PepSwift, 200 µm inner diameter x 25 cm long). The gradient consisted of 2-40% B for 60 min, 40-95% B for 30 min, constant 99% B 15 min and return to 2 % B. The column was re-equilibrated for 15 min at 2% B between runs. The flow rate was set to 0.8 µL/min at 60°C.

The eluate was sprayed using a SilicaTip emitter with 30 μm inner diameter and 360 µm outer diameter (New Objective, Woburn, MA, USA) into a Thermo Finnigan Nanospray Ion Source 1. Standard mass spectrometric conditions for all experiments were spray voltage 3.5 kV, no sheath and auxiliary gas flow; heated capillary temperature, 275 °C; predictive automatic gain control (AGC) enabled, and an S-lens RF level of 60%.

Data were acquired using Xcalibur software (version 3.0.63; Thermo Fisher Scientific, San Jose, CA). The LTQ Orbitrap Velos Pro instrument was operated in positive mode in data-dependent mode using a high-high strategy, meaning that a FT-MS spectrum using the profile mode was followed by an FT-MS2 spectrum. Target resolution in the Orbitrap was set to R = 100,000. In the scan range of m/z 400-2,000, the 5 most intense ions with charge states ≥2 were sequentially isolated and fragmented by HCD (Higher-Energy Collisional Dissociation) with normalized collision energy of 38% and wideband-activation enabled. Ion selection threshold was 1,000 counts for MS/MS with an isolation width m/z = 3. The maximum allowed ion accumulation times were 200 ms for full scans (4 microscans) and 500 ms for HCD-MS/MS measurements (2 microscans) in the Orbitrap analyzer. Target ion quantity for FT full MS was 1×10^6^ and for MS2 was 5×10^5^. Dynamic exclusion was enabled with a repeat count of 1 and exclusion duration of 60 seconds. A lock mass was enabled for accurate mass measurements. Polydimethylcyclosiloxane (m/z, 445.1200025, (Si(CH3)2O))6) ions were used for internal recalibration of the mass spectra.

*Data analysis*

µLC-MS/MS raw files were automatically processed inside ProSight PC software v 4.0 (Thermo Fisher, San Jose) using cRAWler and Xtract algorithms to convert each precursor/fragmentation scan pair into monoisotopic neutral mass values. The process settings were: signal/noise: 2/1, minimum fragment intensity at 100, retaining only the top 5 most intense neutral fragment masses within a 100 Da window.

As previously described^1^, Automated searches were performed using the “Biomarker” search options against a database made via shotgun annotation from the Swiss-Prot *Ovis aries* (sheep) release from the UniProtKnowledgebase release 2018_07. Iterative search tree was designed to begin with high mass accuracy (25ppm at the intact level and 15ppm at the fragment ion level) for monoisotopic precursors. If the top result matched with a E-value of ≤1x10^-6^, the search engine accepted this result as valid and the next search was executed. Subsequent searches used larger intact mass tolerances (average precursors with 2Da mass tolerance and 15ppm at the fragment ion level) if a result of sufficient quality could not be determined initially. A more stringent E-value cutoff of 1x10^−8^ was then applied. An “Absolute mass” search was also performed against the same database using 1000 Da for precursor search window.

For all searches, both delta mass feature and N-terminal post-translation modifications (acetylation and initial methionine cleavage) were considered, and disulfide modifications was deactivated. The same process was iterated in the simple and complex top-down databases.

Computed average mass and isoelectric point (pI) were calculated using ExPASy Compute pI/Mw tool (<https://web.expasy.org/compute_pi/>). They were assigned to a MALDI-TOF peak (an m/z) if the computed mass with PTMs (+/- observed mass difference) was within a ± 0.05% error mass tolerance.

Uncharacterized proteins were mapped to the corresponding *Ovis aries* orthologues by identifying the reciprocal-best-BLAST hits using blastp program (<http://blast.ncbi.nlm.nih.gov/Blast.cgi>).

Gene Ontology and system biology analysis were performed using list of identified proteins. Gene symbols corresponding to these proteins were analyzed for their implication in biological process, molecular functions and pathways using PANTHER database (<http://pantherdb.org>), STRING software (<https://string-db.org>) and MEDLINE database (<https://www.ncbi.nlm.nih.gov/pubmed>).

Also, we compared our dataset with the CASBAH database (<http://bioinf.gen.tcd.ie/casbah/>), which contained a comprehensive list of caspases substrates (downloaded at August 2019, 777 entries), and with the MEROPS peptidase database (<https://www.ebi.ac.uk/merops/index.shtml>) which contained a list of proteolytic enzymes and their substrates (August 2019, 4,000 individual peptidases and inhibitors).

**Bottom-up analysis**

*Electrophoresis and protein digestion*

Heart tissue samples were thawed on ice. For tissue protein extraction, ∼200 mg of sheep heart tissue samples were minced into 2 mm pieces (>3 pieces) and were homogenized in 600 µL of lysis buffer (50 mM Tris, pH 7.4, 0.5% SDS, 1/20 inhibitors of proteases (P2714; Sigma). Samples were homogenized with a sonicator (10 x 20 sec medium 2). After homogenization samples were centrifuged at 11 000 rpm for 90 min at 4°C, and supernatants were stored at −20 °C. Protein concentration was determined using the DC protein assay (BioRad).

Each pool was analyzed (75 µg per lane) on a 4-20% gradient polyacrylamide gel. The gel was stained with Coomassie Blue R-350 and each lane was cut in 20 bands.

As previously described^5^, gel pieces were washed in water: acetonitrile solution (1:1, 5 min) followed by 100% acetonitrile (10 min). Reduction and cysteine alkylation were performed by successive incubation with 10 mM dithiothreitol in 50 mM NH_4_HCO_3_ (30 min, 56 °C), then 55 mM iodoacetamide in 50 mM NH_4_HCO_3_ (20 min, room temperature, in dark). Pieces were then incubated with 50 mM NH_4_HCO_3_ and acetonitrile (1:1, 10 min) followed by acetonitrile (15 min). Proteolytic digestion was carried out overnight using 25 mM NH_4_HCO_3_ with 12.5 ng/μl Trypsin (Sequencing grade, Roche diagnostics, Paris, France). Resultant peptides were extracted by incubation in 5% formic acid (sonicated) with the supernatant removed and saved, followed by incubation in acetonitrile and 1% formic acid (1:1, 10 min) and a final incubation with acetonitrile (5 min), again supernatant was removed and saved. These two peptide extractions were pooled and dried using a SPD1010 speedvac system (Thermosavant, Thermofisher Scientific, Bremen, Germany) and then reconstituted in 20 μL water/1% formic acid (v/v).

*GeLC-MS/MS*

All digested peptide mixtures were separated by on-line nanoLC and analyzed by nano-electrospray tandem mass spectrometry. As previsously described^6^, the experiments were performed Orbitrap Fusion mass spectrometer system (ThermoFisher Scientific) coupled to an Ultimate 3000 RSLC Ultra High-Pressure Liquid Chromatographer (Dionex, Amsterdam, The Netherlands) controlled by Chromeleon Software (version 6.8 SR11; Dionex, Amsterdam, The Netherlands). The peptide mixtures were injected onto a nano trap column (Acclaim C18, 100 μm i.d. x 2 cm length) with a flow of 5 μl.min^-1^ and subsequently gradient eluted with a flow of 300 nl.min^-1^ in an Easy-Spray column (Acclaim PepMap RSLC C18, 2μm, 100A, 75μm x 50cm) from 4 to 40% acetonitrile/0,1% formic acid (v/v) during 90 minutes. Full MS scans were acquired at high resolution (FWMH 120,000) in the Orbitrap analyzer (mass-to-charge ratio (m/z): 400 to 2000), while collision-induced dissociation (CID) spectra were recorded. The mass spectrometer was operated in positive mode in a data-dependent mode to automatically switch between orbitrap-MS and linear trap MS/MS (MS2) acquisition during 3 seconds between master scans.

*Data analysis*

As previously described^7^, the search parameters of Proteome Discoverer 2.1 software (ThermoFisher Scientific, Bremen, Germany) against NCBIprot_mammals database (July 2019) included trypsin as a protease with two allowed missed cleavages and carbamidomethylcysteine, methionine oxidation and acetylation of N-term protein as variable modifications. The tolerance of the ions was set to 10 ppm for parent and 0.8 Da for fragment ion matches. Mascot results obtained from the target and decoy databases searches were subjected to Scaffold software (v 4.8.9, Proteome Software, Portland, USA) using the protein cluster analysis option (assemble proteins into clusters based on shared peptide evidence). Peptide and protein identifications were validated and accepted if they could be established at greater than 95.0% probability as specified by the Peptide Prophet algorithm and by the Protein Prophet algorithm, respectively. Protein identifications were accepted if they contained at least two identified peptides. The False Discovery Rate (FDR) was < 0.01 %.

*Label-free protein quantifications*

For comparative analyses, we applied two independent label-free quantitative methods: 1) the Spectral Counting (SC) using the “Weighed Spectra” method. ; 2) the Extracted-ion chromatogram (XIC) using the Average Precursor Intensity (API) method.

Limits of an average normalized weighted spectra (NWS) of ≥5 and fold change/ratio of ≥2 were included to increase validity of any comparisons made.

Due to preliminary individual nanoLC-MS/MS analysis without pre-fractionation by SDS-PAGE (total extract included in one band), we evaluated the biological reproducibility linked to individual variability (considering N=5 samples per condition) and the technical repetability linked directly to the nanoLC-MS methodology (considering 3 technical replicates). By this way, we calculated the coefficients of variance (CVs) for proteomic experiments from API data. Overall, biological CVs did not exceed 8% and 10%, 15% and 11% in “near” and “far” regions for control group and electrical shock group respectively while technical CVs never exceeded 6.45%. In this way, the 4 populations were considered as homogeneous and proper for large bottom-up proteomic experiments using GeLC-MS/MS strategy from pooled and fractionated samples by SDS-PAGE.

*Gene Ontology, localisation and network analysis*

Gene Ontology and system biology analysis were performed using list of identified and quantified proteins. Gene symbols of these proteins were analyzed for their implication in biological processes, molecular functions and pathways using PANTHER database (<http://pantherdb.org>), STRING software (<https://string-db.org>) and MEDLINE database (<https://www.ncbi.nlm.nih.gov/pubmed>).

*Immunoblot analysis*

Protein concentration was determined using the DC protein assay (BioRad). Protein extracts from each individuals were migrated (10μg of proteins per lane) on a SDS-PAGE 8–16% gradient gel and blotted on a nitrocellulose membrane using the Trans-Blot Turbo Transfer System (BioRad, Marnes-la-Coquette, France). The membranes were stained with Ponceau S solution (5 min at room temperature, gentle shaking) and scanned with Image Scanner (Amersham Biosciences, GE Health-care LifeSciences) to check the homogeneous loading among lanes and for normalization (see below). Membranes were blocked in 5% (w/v) milk powder diluted in TBS-T (Tris-buffered saline with 1% (v/v) Tween 20, gentle shaking, 4 °C, overnight) and then incubated with the primary antibody diluted at 1:200 for myosin light chain II (MYL2 (7C9): sc-517244, Santa Cruz Biotechnology, Dallas, USA) and 1:100 for glyceraldehyde 3-phosphate dehydrogenase (GAPDH (D-6): sc-166545, Santa Cruz Biotechnology Inc., Dallas, Texas, USA) (gentle shaking, 4 °C, overnight). Blots were finally incubated with fluorescent secondary antibody IRDye 800CW anti-Mouse IgG (gently shaking, 37°C, darkness, 1h) diluted at 1:5,000 before revelation with infrared scanner Odyssey CLx (LI-COR Biotechnology, Lincoln, USA). Protein fluorescent signals were analyzed by Image Studio software (LICOR Biotechnology, Lincoln, USA). The bands were quantified afterwards using ImageQuantTL (GE Healthcare LifeSciences).

Three biological replicates were performed for each antibody and each condition. To normalize the data, Ponceau S staining was used, as previously described^28^. Briefly, the whole lanes were quantified by densitometry using the TotalLab Quant software (version11.4, TotalLab, Newcastle upon Tyne, UK). Then the electrical shock groups were normalized with the control group.

Mean fold changes of normalized volume (fluorescence) were calculated for the two proteins of interest. To compare control and electrical shock groups, a non-parametric Mann Whitney statistical test was used.

**REFERENCES**

1. Labas V, Teixeira-Gomes A-P, Bouguereau L, Gargaros A, Spina L, Marestaing A, Uzbekova S. Intact cell MALDI-TOF mass spectrometry on single bovine oocyte and follicular cells combined with top-down proteomics: A novel approach to characterise markers of oocyte maturation. *J Proteomics*. 2018;175:56–74.

2. Uzbekova S, Elis S, Teixeira-Gomes A-P, Desmarchais A, Maillard V, Labas V. MALDI Mass Spectrometry Imaging of Lipids and Gene Expression Reveals Differences in Fatty Acid Metabolism between Follicular Compartments in Porcine Ovaries. *Biology (Basel)*. 2015;4:216–236.

3. Soler L, Labas V, Thélie A, Grasseau I, Teixeira-Gomes A-P, Blesbois E. Intact Cell MALDI-TOF MS on Sperm: A Molecular Test For Male Fertility Diagnosis. *Molecular & Cellular Proteomics : MCP*. 2016;15:1998.

4. Soler L, Labas V, Thélie A, Teixeira-Gomes AP, Grasseau I, Bouguereau L, Blesbois E. Data on endogenous chicken sperm peptides and small proteins obtained through Top-Down High Resolution Mass Spectrometry. *Data Brief*. 2016;8:1421–1425.

5. Gundry RL, White MY, Murray CI, Kane LA, Fu Q, Stanley BA, Van Eyk JE. Preparation of Proteins and Peptides for Mass Spectrometry Analysis in a Bottom-Up Proteomics Workflow. *Curr Protoc Mol Biol*. 2009;CHAPTER:Unit10.25.

6. Labas V, Grasseau I, Cahier K, Gargaros A, Harichaux G, Teixeira-Gomes A-P, Alves S, Bourin M, Gérard N, Blesbois E. Qualitative and quantitative peptidomic and proteomic approaches to phenotyping chicken semen. *J Proteomics*. 2015;112:313–335.

7. Marie P, Labas V, Brionne A, Harichaux G, Hennequet-Antier C, Nys Y, Gautron J. Data set for the proteomic inventory and quantitative analysis of chicken uterine fluid during eggshell biomineralization. *Data Brief*. 2014;1:65–69.
