# Supplementary figures and images for: Acute pathophysiological myocardial changes following intra-cardiac electrical shocks using a proteomic approach in a sheep model
Source: Sci Rep. 2020 Nov 20;10:20252. doi: 10.1038/s41598-020-77346-x (PMC7679418; doi:10.1038/s41598-020-77346-x)

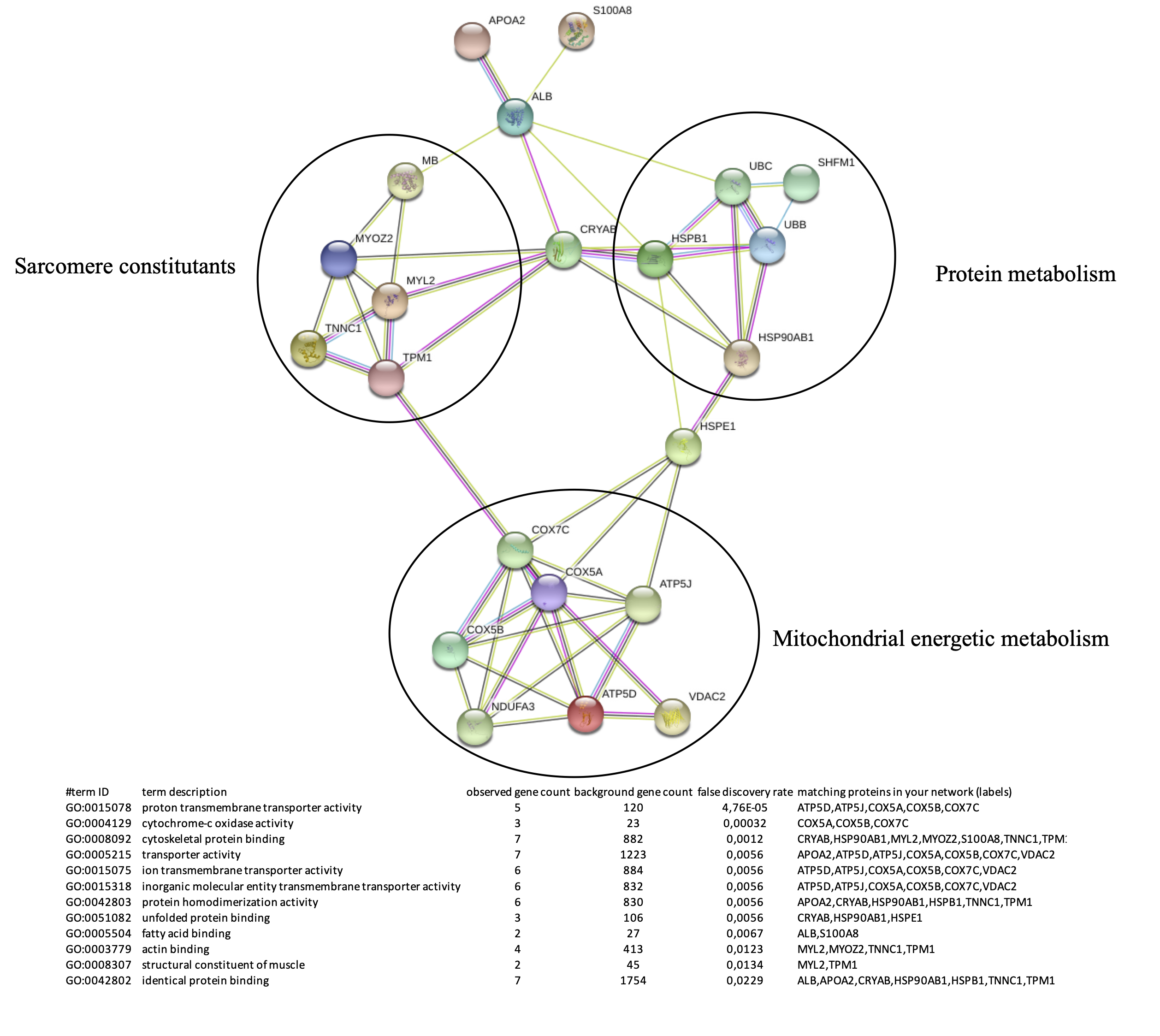

Supplement: Supplementary file 6 — Supplementary Figure 1. [file 41598_2020_77346_MOESM6_ESM.tiff]

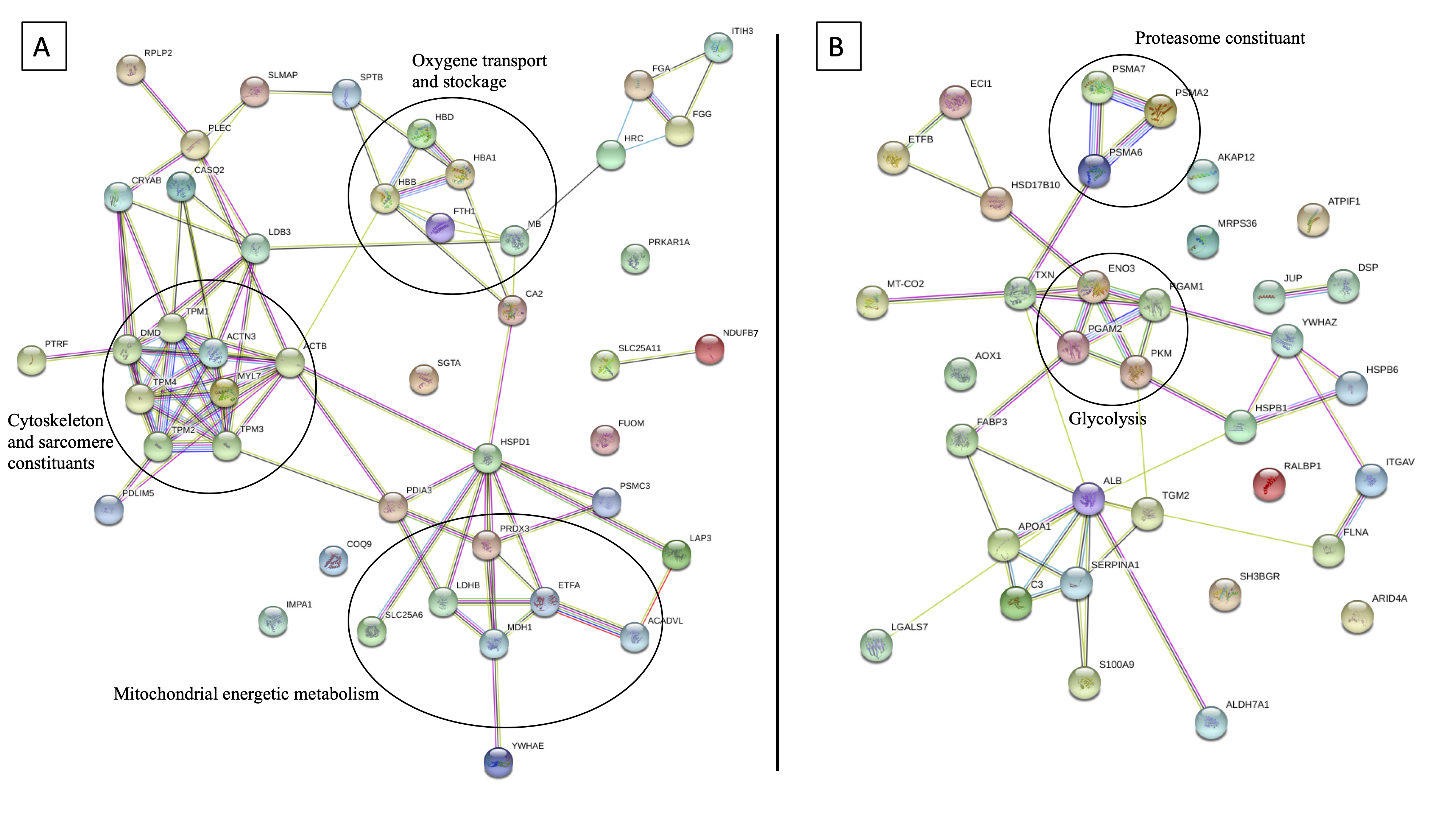

Supplement: Supplementary file 7 — Supplementary Figure 2. [file 41598_2020_77346_MOESM7_ESM.tiff]

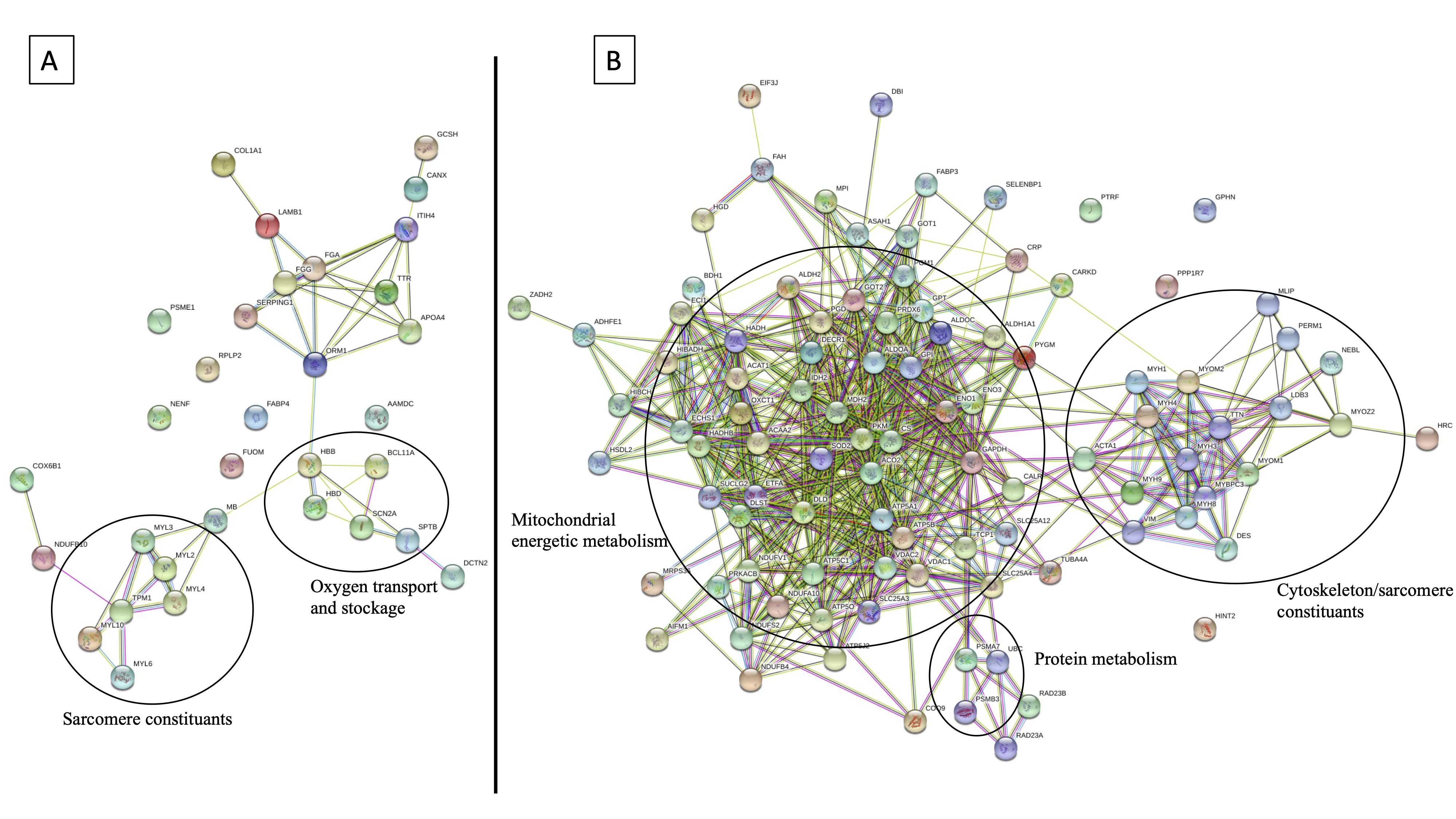

Supplement: Supplementary file 8 — Supplementary Figure 3. [file 41598_2020_77346_MOESM8_ESM.tiff]
