# Supplementary material for: Acute pathophysiological myocardial changes following intra-cardiac electrical shocks using a proteomic approach in a sheep model
Source: Sci Rep. 2020 Nov 20;10:20252. doi: 10.1038/s41598-020-77346-x (PMC7679418; doi:10.1038/s41598-020-77346-x)
Supplement: Supplementary file 9 — Supplementary Legends. [file 41598_2020_77346_MOESM9_ESM.docx]

**Supplemental figure 1 –** **Functional analysis of TD-HR MS.** Representation of links between identified proteins corresponding to an unique gene with the TD-HR MS with a STRING interaction network in the upper part (<https://string-db.org>). Gene Ontology (GO) molecular functions terms associated are represented below using PANTHER database (<http://pantherdb.org>).

**Supplemental figure 2 – Functional analysis of bottom-up “near” region.** Representation of links between decreased proteins (A) and increased proteins (B) in the “near” region following electrical shocks, with a STRING interaction network (<https://string-db.org>).

**Supplemental figure 3 – Functional analysis of bottom-up “far” region.** Representation of links between decreased proteins (A) and increased proteins (B) in the “far” region following electrical shocks, with a STRING interaction network (<https://string-db.org>).
